# Optically Generated Ultrasound for Intracoronary Imaging

**DOI:** 10.3389/fcvm.2020.525530

**Published:** 2020-10-14

**Authors:** Callum D. Little, Richard J. Colchester, Sacha Noimark, Gavin Manmathan, Malcolm C. Finlay, Adrien E. Desjardins, Roby D. Rakhit

**Affiliations:** ^1^Department of Cardiovascular Medicine, Royal Free NHS Foundation Trust, London, United Kingdom; ^2^Wellcome-Engineering & Physical Sciences Research Council (EPSRC) Centre for Interventional and Surgical Sciences, London, United Kingdom; ^3^Department of Medical Physics and Bioengineering, University College London, London, United Kingdom; ^4^William Harvey Cardiovascular Research Institute, Queen Mary University of London and Barts Health Centre London, London, United Kingdom

**Keywords:** optical ultrasound, OPUS, optoacoustics, imaging, intravascular ultrasound, IVUS

## Abstract

Conventional intravascular ultrasound (IVUS) devices use piezoelectric transducers to electrically generate and receive US. With this paradigm, there are numerous challenges that restrict improvements in image quality. First, with miniaturization of the transducers to reduce device size, it can be challenging to achieve the sensitivities and bandwidths required for large tissue penetration depths and high spatial resolution. Second, complexities associated with manufacturing miniaturized electronic transducers can have significant cost implications. Third, with increasing interest in molecular characterization of tissue *in-vivo*, it has been challenging to incorporate optical elements for multimodality imaging with photoacoustics (PA) or near-infrared spectroscopy (NIRS) whilst maintaining the lateral dimensions suitable for intracoronary imaging. Optical Ultrasound (OpUS) is a new paradigm for intracoronary imaging. US is generated at the surface of a fiber optic transducer via the photoacoustic effect. Pulsed or modulated light is absorbed in an engineered coating on the fiber surface and converted to thermal energy. The subsequent temperature rise leads to a pressure rise within the coating, which results in a propagating ultrasound wave. US reflections from imaged structures are received with optical interferometry. With OpUS, high bandwidths (31.5 MHz) and pressures (21.5 MPa) have enabled imaging with axial resolutions better than 50 μm and at depths >20 mm. These values challenge those of conventional 40 MHz IVUS technology and show great potential for future clinical application. Recently developed nanocomposite coating materials, that are highly transmissive at light wavelengths used for PA and NIRS light, can facilitate multimodality imaging, thereby enabling molecular characterization.

## Introduction

Intravascular imaging has the ability to provide invaluable anatomical information to facilitate the treatment of coronary artery disease (1). Additionally, molecular compositional analysis of atheroscletoric plaque disease may help to identify targets for intervention (2). The two leading technologies in this field for microstructural imaging are Optical Coherence Tomography (OCT) and Intravascular Ultrasound (IVUS). OCT images are obtained using broadband near-infrared light (wavelengths typically in the vicinity of 1,310 nm), with reflections from tissue detected interferometrically. High axial (10 μm) and lateral (20 μm) resolutions allow for assessments of both vascular endothelium and plaque structural components, albeit with limited tissue penetration (typically <2 mm) (3). IVUS is a modality analogous to OCT, in which ultrasonic reflections from tissue are detected. Typical commercial IVUS imaging catheters operate with transducer frequencies centered at around 40 MHz, providing an axial resolution of 100–150 μm, a lateral resolution of 200 μm and a tissue penetration of 4–8 mm (3). IVUS has established roles in sizing vessels, detecting calcium, and guiding optimal stent expansion. IVUS has seen exciting technological advancements in recent years, including the use of dual frequency probes to allow for high resolution imaging (4) and combinations with near-infrared spectroscopy (NIRS) (5) or photoacoustics (PA) (6) to provide hybrid imaging with molecular compositional analysis.

With contemporary IVUS devices, ultrasound (US) is generated and received electrically, using piezoelectric transducers. Whilst this electronic platform is well-established within the field of interventional cardiology, there are limitations that preclude its broader clinical use. First, with very small elements (e.g., diameter <150 μm), it can be challenging to achieve adequate sensitivity and bandwidth for high penetration depth and high resolution tissue imaging. Second, the complexities associated with fabricating and electrically connectorising broadband piezocomposite transducers can result in high manufacturing costs. Third, the broader applicability of electronic interventional devices in the coronary catheterization suite is challenged by sensitivities to electromagnetic interference and lack of MRI compatibility (7).

Optical ultrasound (OpUS) imaging probes, in which transmission and reception are both performed with light, are emerging as alternatives to their electrical counterparts. They offer several key advantages, including the potential to generate and detect the broadband US fields (tens of MHz) required for high resolution intravascular imaging (8) and immunity to EM interference. Moreover, optical fibers used for ultrasound transmission and reception can provide the required level of miniaturization for minimally invasive use and have costs that lend themselves to disposable devices. Furthermore, the use of optical fibers allows for the integration of complementary imaging and therapeutic modalities without compromising the device size or performance (9). In this review, we describe the application of OpUS to coronary imaging, including preclinical data acquired using this technology, and future translational applications.

### Optical Ultrasound Generation and Reception

With optical ultrasound (OpUS) the generation of US occurs via the photoacoustic effect at the surface of a fiber optic transducer (8), wherein pulsed or modulated excitation light is absorbed in a coating and converted to thermal energy. The transient heat rise leads to a corresponding pressure rise which propagates as an ultrasound wave. The bandwidth of this wave depends on the temporal characteristics of the excitation light. In general, the bandwidth can be increased by decreasing the duration of excitation light pulses; however, in practice, these increases are limited by frequency-dependent ultrasound attenuation within the coating (10) and within blood and vascular tissue (11). To achieve efficient optical-US transduction, a material with a high optical absorption coefficient and a high thermal expansion coefficient is desirable. Studies to date have highlighted the efficacy of composite materials that comprise optical absorbers integrated within elastomers. With these considerations in mind, several nanocomposite materials have been explored, including carbonaceous materials (8), metallic nanoparticles (9), and organic pigments (9). Carbonaceous materials including carbon black (12), carbon nanofibers (13), candle soot (14), carbon nanotubes (15), and graphene (16) have high optical absorption across the visible and near-infrared wavelength ranges. Metallic nanoparticles such as gold, exhibit a relatively narrow optical absorbing window. Organic pigments such as crystal violet can display poor photostability with repeated usage causing photobleaching with loss of acoustic conversion efficiency (9). Noimark et al. showed for the first time that functionalized multiwalled carbon nanotube-polydimethylsiloxane (PDMS) composites can give rise to pressures of 21.5 MPa at the coating surface (the highest recorded pressure from a fiber optic transmitter, to our knowledge) and bandwidths of 39.8 MHz (15). These high pressures enable high imaging penetration depths, and the broad bandwidths give rise to high axial resolution. A small coating thickness can be important to minimize acoustic attenuation within nanocomposite materials (10). To this end, several methods have been explored for depositing nanocomposite materials onto the distal surface of the fiber optic transducer, including spin-coating (17), electrospinning (18), and dip-coating (15). With the latter method, a “bottom up” approach typically involves coating the substrate with an optical absorber and a subsequent polymer overcoat (for instance, PDMS). Control of the thickness of the nanocomposite region comprising the optical absorber and the polymer, and the total thickness of the polymer, is determined by both the viscosity of the material, which can be altered through the use of solvents, and the dipping speed of the optical fiber.

**Figure 1:**
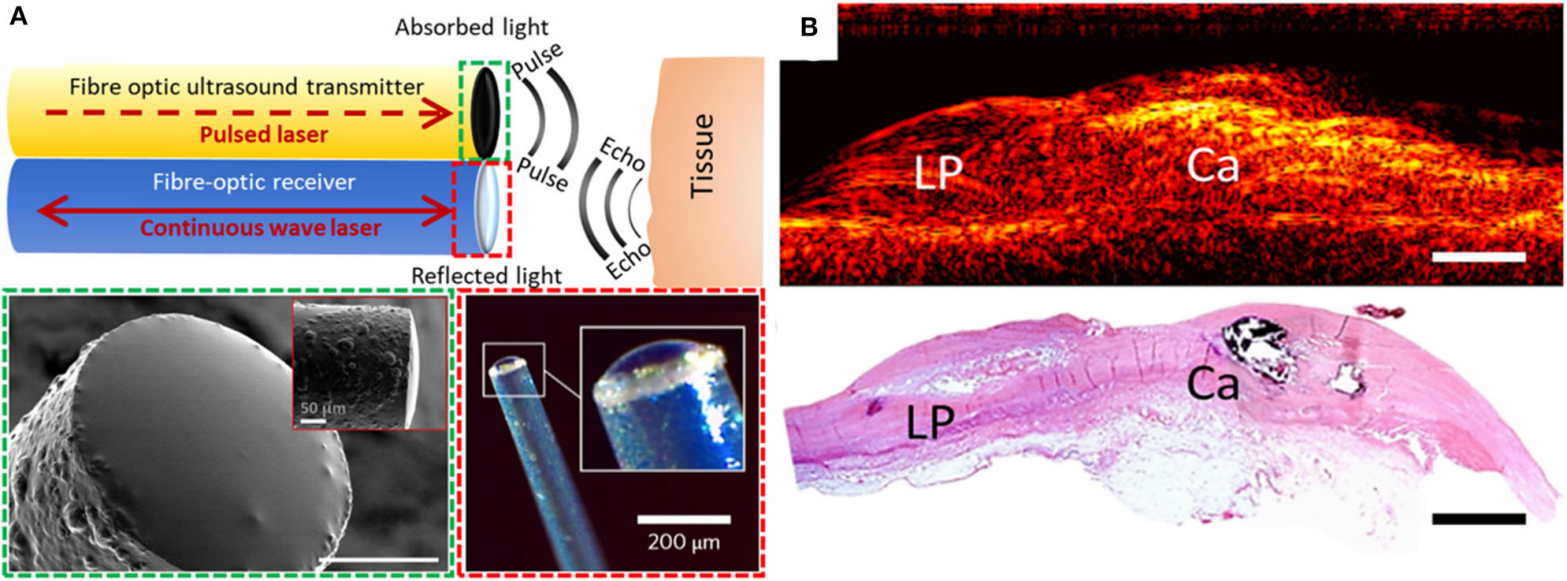
**(A)** Generation and reception of OpUS. The schematic (top) includes both a transmitter and a receiver. Lower-left inset (dashed green box): scanning electron microscopy image of the transmitter fiber-tip coated with nanocomposite (scale bar: 50 μm) (15). Lower-right inset (dashed red box): optical microscopy of the fiber-optic receiver (19). **(B)** 2D longitudinal M-mode OpUS imaging of *ex-vivo* human coronary artery tissue with a lipid pool (LP), calcification (Ca), and corresponding histology (haematoxylin and eosin staining) (scale bars: 2 mm).

To allow for real-time pulse-echo US imaging, fiber-optic transmitters have been be paired with high-finesse Fabry-Pérot fiber-optic receivers [(19); Figure 1A]. These receivers, fabricated on the distal ends of optical fibers, comprise an epoxy dome with high-reflectivity coatings on both the planar and domed surfaces. When an ultrasound wave impinges on the dome, it causes nanometer-scale deformations in the dome surface. These deformations are measured using laser interferometry. Such devices are capable of measuring pressures lower than 100 Pascals, making them highly sensitive to ultrasound reflections and ideally suited to minimally invasive imaging. In contrast to piezoelectric receivers, whose sensitivities fall off with decreasing element size, fiber optic receivers can maintain their sensitivity even at scales of tens or hundreds of micrometers. Their small lateral dimensions (<250 μm outer diameter) enable integration into intracoronary imaging devices, and their large bandwidths yield high imaging performance [e.g., axial resolution better than 60 μm and cm-scale imaging depths without temporal averaging (20)]. Several other fiber optic reception technologies are promising for intracoronary imaging, including fiber Bragg gratings (21–23) and microring resonators (24, 25).

With OpUS, high bandwidths and pressures have enabled imaging with resolutions better than 50 μm and tissue penetration depths >20 mm (20). These values challenge those of conventional 40 MHz IVUS technology and show great potential for future clinical application. As initial demonstrations of the viability of OpUS for *in-vivo* clinical imaging, forward-viewing configurations have been used. With these configurations, ultrasound was transmitted ahead of the optical fiber, in a direction colinear with the optical fiber axis. Axial and lateral resolutions of <60 and <90 μm, respectively, were achieved (26). A forward-viewing probe that was integrated within a transseptal puncture needle was used to obtain the first *in vivo* intracardiac images with OpUS (27). Recently, the first OpUS images of *ex-vivo* human coronary tissue samples (Figure 1B) were acquired and compared to histology. Numerous features of atherosclerotic plaque were identifiable, including a lipid pool, a calcified nodule, and the different layers of the vessel wall. Several other configurations relevant to intracoronary imaging have subsequently been developed, including side-viewing rotational imaging (20) and hybrid multimodality imaging (9).

### OpUS Structural Imaging

**Figure 2:**
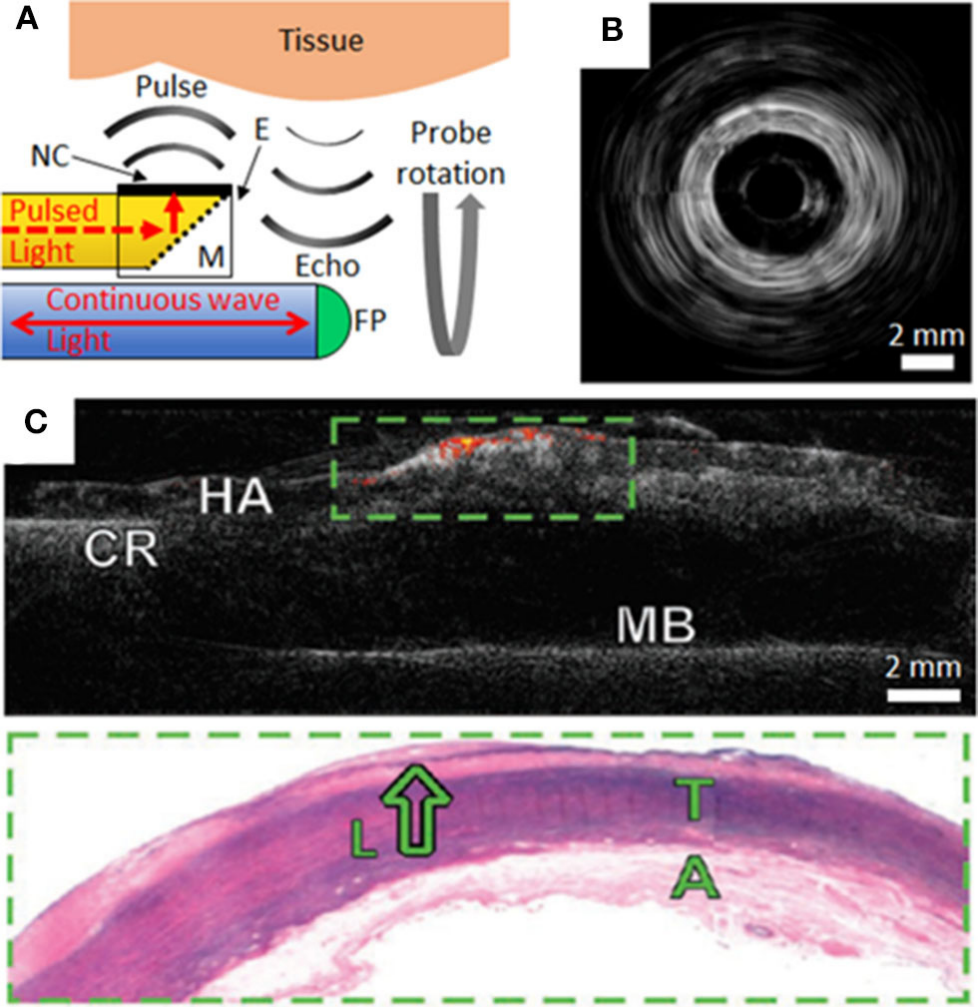
**(A)** Schematic of side-viewing optical ultrasound imaging probe with pulsed excitation light transmitted along an optical fiber (yellow), reflected at a mirror (M) and absorbed at the nanocomposite (NC) coating surface of an epoxy-based (E) optical transducer housing. Ultrasound pulses reflect from tissue and the echoes are received through interferometric interrogation of the Fabry-Pérot (FP) cavity at the distal end of the receiving optical fiber (blue). The probe is rotated about its axis to achieve circumferential imaging. **(B)** Rotational optical ultrasound images obtained within an *ex-vivo* swine carotid artery (20). **(C)** Co-registered optical ultrasound (gray-scale) and photoacoustic images of human aortic tissue (HA) using a gold nanoparticle/PMDS composite transmitter. Areas of lipid are displayed as a color signal. The tissue was secured to a cork ring (CR) and metal base (MB) for imaging. Corresponding histology of the green boxed area highlights a lipid pool (L), tunica media (T) and adventitia (A) (9).

Commercial intravascular imaging systems can employ rotational pullbacks of side-viewing probes in order to achieve cross-sectional imaging (with phased-array probes as alternatives). An analogous rotational implementation with OpUS can be realized with ultrasound transmitted perpendicular to the device axis. In a recent probe, perpendicular ultrasound transmission was made possible with an optically-absorbing nanocomposite coating extending perpendicular to the axis of the optical fiber that transmitted excitation light, in conjunction with a 45° mirror (Figure 2A). This optical fiber was connected to a rotary junction in order to allow for circumferential imaging, whilst the omnidirectional fiber-optic receiver remained stationary. The configuration of this imaging device had dimensions suitable for intracoronary imaging, with a maximum lateral dimension <1.25 mm at the distal tip. Image acquisition occurred with a frame rate of 5 frames per second. The imaging fidelity of the device was investigated with *ex-vivo* swine carotid tissue [(20); Figure 2B]. The broad bandwidths achieved (−6 dB bandwidth of 31.3 MHz), in conjunction with depth-dependent digital frequency filtering, allowed for both high axial resolutions at shallow depths and deep tissue visualization. Axial and lateral resolutions at a distance of 3 mm from the probe were superior to 50 μm and 15°. This device sets the stage for future devices with rotational lengths and encapsulation suitable for *in vivo* intracoronary imaging.

### Multi-Modality US and Photoacoustic Imaging

Recently there has been intense interest in multi-modality intravascular imaging probes that provide complementary structural and molecular information. These probes include combinations such as US with photoacoustic (PA) imaging (6, 28) or with near-infrared spectroscopy (NIRS) (5, 29). A potential strong advantage of OpUS probes, as compared to their electronic counterparts, is that the optical fibers used to generate and receive ultrasound can also be used to transmit light for PA and NIRS. These efficiencies, which can be achieved through the use of nanocomposite fiber tip coatings that are absorbing at certain wavelengths and transmissive at others, could be valuable both for achieving high levels of miniaturization and cost reductions. In a study by Noimark and Colchester et al., gold nanoparticle composites and organic dye composites, applied to the distal ends of optical fibers within an imaging probe, were used to generate ultrasound for OpUS (532 nm wavelength) and to transmit light for PA imaging (1,210 nm) from a single optical fiber (9). Co-registered ultrasound and photoacoustic images of an *ex-vivo* diseased human aorta were acquired. PA imaging provided molecular contrast for lipid rich coronary plaque, which was overlaid on the acquired ultrasound images (Figure 2C). Whilst the use of multi-modality OpUS for intracoronary imaging is at an early stage, there are strong indications that it could allow for measuring the plaque burden, which in turn could be valuable for guiding stent placement and for improving our understanding of the pathophysiology of coronary artery disease.

## Discussion

Direct comparison of OpUS to established intracoronary imaging devices is challenging at present due to a lack of *in-vivo* clinical data. Whilst detection of lipid and calcium by OpUS has been demonstrated (Figure 1B), further work is required to assess the efficacy of this technology for accurately sizing vessel diameter, luminal area, optimizing stent expansion, and detection of high risk atherosclerotic plaque morphological features including fibrous cap thickness, neovascularisation and macrophage infiltration. Nevertheless, OpUS potentially offers several prominent benefits over current generation electronically generated ultrasound. The axial resolution could be enhanced by the use of modulated excitation light that emphasizes higher ultrasound frequencies. Improvements to the lateral resolution could be achieved with curved transducers to achieve a focussed beam and with shorter excitation light pulses to increase the central frequency, and potentially with deep-learning beamforming for sub-sampled data (30). In order to improve image quality further refinements in image post-processing are possible, including complex filtering and reconstruction approaches, as well as adaptive frequency filtering to allow discrimination between acoustically dissimilar tissues (31–35). Significant increases in image acquisition rate are readily achievable. The rotational OpUS setup demonstrated to date (20) was limited to 5 frames per second by the data transfer rate and computation times, but further engineering developments with existing excitation lasers could potentially increase imaging rates to over 100 frames per second without compromising lateral resolution. To enable more rapid circumferential imaging for clinical translation, mechanical elements such as torque coils could be incorporated into the imaging probes, as used with OCT catheters. It is envisaged that lubricating fluid will be necessary to reduce frictional forces generated by the rotational components and to facilitate ultrasound coupling. Looking beyond the paradigm of rotating a single optical transducer, a phased array analogous to solid-state IVUS systems could be envisaged; however, it is likely to be challenging to achieve a high density of optical transducer elements within a coronary imaging device without compromising the device profile or deliverability. It is conceivable that spatial light modulators for controlling the propagation of laser light through multimode optical fibers (36) could be used in this context. A forward-looking volumetric probe analogous to electronic IVUS versions (37) could be envisaged; however, the spatial resolution would be highly dependent on the geometry and spatial configuration of the transducer elements.

Looking beyond aforementioned probes for vascular imaging that have been demonstrated at a pre-clinical level, there are uncharted frontiers in which OpUS probes could be extended to include new capabilities. They include enhanced diagnosis via pairing with OCT, photoacoustic imaging (38), and near-infrared fluorescence (NIRF) (39); additionally, they include therapeutic monitoring during atherectomy (40) and device tracking with the inclusion of fiber optic shape sensing (41). Speculatively, the inclusion of OCT could be enabled with a double-clad optical fiber, with ultrasound reception and OCT performed with light transmitted along the single-mode inner core and ultrasound transmission with the multi-mode outer core. Optically-generated ultrasound represents an exciting development in the field of intravascular imaging. Whilst many steps along the translational path remain, there is strong potential to unite many modalities into a single fiber optic probe that could have broad applicability in cardiovascular medicine and beyond.

## Author Contributions

CL, RC, and AD co-wrote the paper. SN provided advice on descriptions of the nanocomposite materials. GM, MF, and RR provided advice on the clinical applications of OpUS. All authors contributed to the article and approved the submitted version.

## Conflict of Interest

AD, RC, and MF are shareholders of Echopoint Medical Ltd. The remaining authors declare that the research was conducted in the absence of any commercial or financial relationships that could be construed as a potential conflict of interest.
